# The Impacts of Working With Victims of Sexual Violence: A Rapid Evidence Assessment

**DOI:** 10.1177/15248380211016024

**Published:** 2021-05-18

**Authors:** Ioana M. Crivatu, Miranda A. H. Horvath, Kristina Massey

**Affiliations:** 1 Department of Psychology, 4907 Middlesex University, United Kingdom; 2 School of Law, Criminal Justice and Policing, 2238 Canterbury Christ Church University, United Kingdom

**Keywords:** practitioner, professional, review, compassion fatigue, vicarious trauma, sexual violence, burnout

## Abstract

**Aim:**

Supporting clients who have experienced trauma can lead to trauma symptoms in those working with them; workers in the sexual violence field are at heightened risks of these. This article collated and critically appraised papers, published from 2017 onward, in the area of people assisting victims of sexual violence. It explores the impacts and effects the work has on them, their coping and self-care mechanisms, and organizational support offered to them.

**Design:**

A question-based rapid evidence assessment with a triangulated weight of evidence approach was used. Academic and nonacademic databases were searched. Twenty-five papers were included for analysis based on the inclusion/exclusion criteria.

**Results:**

Most studies were of medium to high methodological quality. Negative impacts included trauma symptoms, disrupted social relationships, behavioral changes, and emotional and psychological distress. Ability to manage negative impacts was influenced by overall organizational support, availability of training, supervision and guidance, workloads and caseload characteristics, individual characteristics, and their coping and self-care mechanisms. Positive impacts included empowering feelings, improved relationships, compassion satisfaction, and posttraumatic growth.

**Conclusions:**

Impacts are significant. Support at work and in personal life increases staff’s ability to cope and find meaning in their role. Implications for research and practice are discussed.

The “costs” of working with trauma are widely recognized (Hesse, 2002; Maslach, 2003). Research has mostly focused on professionals^1^ working with high-risk clients or situations such as firefighters (Jahnke et al., 2016), ambulance personnel (Kang et al., 2018), substance abuse counselors (Cosden et al., 2016), social workers (Bride, 2007), and police officers and nurses (Bakker & Heuven, 2006; Burke, 1994; Martinussen et al., 2007). The negative impacts most commonly found in trauma professionals include secondary traumatic stress (STS; Figley, 1995), vicarious traumatization (VT; McCann & Pearlman, 1990), compassion fatigue (CF; Figley, 1996, 2002b), and burnout (Figley, 2002a; Maslach, 2003; Maslach & Leiter, 2006).

It may be that when compared with other professionals working with trauma, professionals who assist victims of sexual violence are vulnerable to developing more severe VT symptoms (Cunningham, 2003). Their vulnerability could increase with higher levels of exposure (Brady et al., 1999). This could possibly be due to sexual violence survivors generally experiencing more severe and more long-lasting trauma than survivors of other adversities such as combat or physical violence (Kessler et al., 1995, 2017). The aftermath for victims of sexual violence is well established and includes posttraumatic stress disorder (PTSD), depression, anxiety, dissociation, substance abuse, self-harm, and suicidal ideation (Briere & Jordan, 2004; Mason & Lodrick, 2013; Ullman, 2016), with many of them having preexisting mental health difficulties which exacerbate the trauma (Kessler et al., 2017; Manning et al., 2019). Thus, professionals who work with them play a crucial role in providing help and support to highly traumatized and complex clients, while having to take into consideration an array of difficulties, as well as consistently hearing about traumatic experiences or seeing its effects.

Due to the nature of the trauma they are exposed to, professionals who specialize in working with sexual violence (PSWSV) can experience elevated levels of stress, CF, and VT, often meeting clinically diagnosable levels of PTSD (Baird & Jenkins, 2003; Brady et al., 1999; Choi, 2011). These impacts result in anxiety, depression, intrusive thoughts, alienation and avoidance, feeling of helplessness and hopelessness, and disrupted social relationships (Blair & Ramones, 1996). Clemans (2004) found that employees working in a rape crisis center experienced secondary traumatization and other negative effects such as headaches, stomachaches, panic attacks, and increased anxiety, as well as hypervigilance and overprotection in their parenting styles. Furthermore, due to having to navigate the complex criminal justice system, as well as the high volume of clients, staff often feel overwhelmed and experience frustration and burnout (Baird & Jenkins, 2003). Moreover, supporting victims of sexual violence can impact professionals’ intimate sexual life (Rizkalla et al., 2017) and possibly alter their sexual identities in ways similar to those experienced by the victim themselves (Sabatini Gutierrez, 2018).

The nature and degree of the impacts experienced are dependent on situational factors, such as workload, and on individual factors, such as professionals’ ability to deal with the effects through the use of self-care and coping mechanisms (Pearlman & Saakvitne, 1995). Coping mechanisms are cognitive and behavioral strategies which an individual uses during stressful situations to manage themselves and their emotions (Folkman & Moskowitz, 2004). Utilizing coping strategies promotes resilience, an adaptive state that allows individuals to “bounce back” and deal with long-term stressful situations (Luthar & Cicchetti, 2000; Smith et al., 2010). Resilience is a vital characteristic of professionals working with trauma clients and sexual violence victims in particular (Howard et al., 2015; C. M. McCann et al., 2013; Pack, 2013). Recent research carried out with members of the Faculty of Forensic and Legal Medicine in the United Kingdom found that their individual coping mechanisms and resilience levels were important predictors for the levels of psychological distress suffered (Horvath & Massey, 2018). However, types of coping strategies employed are also important, with avoidance-based ones increasing CF levels and adaptive ones protecting against it (Zeidner et al., 2013). Other factors such as age, gender, years of experience, personality, levels of organizational support, including informal and formal supervision, social support, and having a history of trauma can impact on the effects of working with trauma (Bride et al., 2009; Elwood et al., 2011). For example, having personally experienced adverse situations that are presumed traumatizing can increase professionals’ vulnerability to VT, whereas perceived organizational support and receiving regular supervision reduces its negative impacts (Vrklevski & Franklin, 2008).

It is vital to acknowledge the impacts working with victims of sexual violence may have on staff’s personal and professional life and to take actions to minimize them not only for their welfare but also for the quality of their work and the well-being of their clients. Workplaces have a duty of care to attend to the mental and physical well-being of their professionals (Robinson, 2018). Additionally, the need for specialist help may be at an all-time high given the high numbers of people reporting sexual violence (Office for National Statistics, 2020).

This rapid evidence assessment (REA) reviewed recent publications investigating the impacts and effects working with victims of sexual violence has on professionals, their coping and self-care mechanisms, and the organizational support offered to them. The research questions were as follows:

**Research Question 1:** What are the effects of working with victim-survivors of sexual violence on professionals’ well-being?
**Research Question 2:** What factors influence PSWSV’s ability to deal with the impacts of their work?
**Research Question 3:** What can PSWSV and the organizations they work for do to minimize the impacts of working with victims of sexual violence and facilitate professionals’ well-being?

## Method

### Design

A question-led REA methodology was adopted as it allows for conducting a comprehensive and exhaustive search of the literature within a given time frame, outlining and critically evaluating the available publications on a specific topic, identifying papers of poor quality, and providing an overview of current evidence (Davies, 2003). Compared to a systematic review, which remains the method of choice for most literature reviews, an REA is conducted in a shorter period of time, thus better reflecting the most recent publications in a field, and it is particularly useful for policy makers and health care providers as it informs not only on current outcomes in a specific area but also on the strength, quantity, and quality of the findings (Varker et al., 2015).

### Procedure

#### Inclusion and exclusion criteria

The following search parameters were used to bring together all viable international evidence and to give equal considerations to all methodologies: materials published between January 1, 2017, and January 31, 2020, excepting in press/preparation or seminal work; materials involving professionals in any capacity working with sexual violence victims; publications discussing the impacts and effects the work has on professionals working with sexual violence victims; publicly available academic and nonacademic publications, including research, reviews and meta-analyses, doctoral theses, dissertations and reports; publications in the English language; any jurisdiction and all research methods. This REA focused on literature published in the last 3 years to accommodate the most current international changes in sexual violence law, such as expansions of definitions of sexual violence, or the criminalization of previously acceptable sexually violent practices in Middle Eastern, South Asian, Latin American, and North African countries (United Nations [UN] Women, 2017). Across the globe, such changes have instigated noteworthy and fast developing improvements in the range, availability, and implementation of professional support available to victims (UN Women, 2017; Vandenberghe et al., 2018).

Any publications about professionals working with generally traumatized and/or abused clients without specifying that sexual violence was part of it were excluded. Publications were also excluded if they discussed PSWSV without mentioning how the work impacted them or what coping mechanism they may have used.

#### Search strategy

In order to maintain scope and rigor on the research questions, 67 keywords that fell into three broad categories (professional group, impact, and intervention) were generated and utilized as search terms. The terms in the *professional group* described job roles likely to include work with victims of sexual violence (Daniels, 2016). The *impact* category included well-established probable consequences of working with traumatized clients (Baird & Jenkins, 2003; Brady et al., 1999; Choi, 2011). Lastly, the *intervention* category included terms describing the most common forms of available support for these professionals (Choi, 2011; Clarke, 2011) as well as being a research aim of this REA. Three search strings were created using all possible combinations of the search terms. Table 1 presents the keywords and search categories used.

**Table 1.** Search Categories and Keywords.

Professional Group	Impact	Intervention
Independent Sexual Violence Advocate^a^ Sexual Violence Professional^a^ Counsellor^a^ Crisis worker^a^ Forensic physician^a^ Forensic doctor^a^ Forensic nurse^a^ Police^a^ Clinical psychologist^a^ Psychologist^a^	Trauma^a^ Vicarious trauma^a^ Secondary trauma^a^ Secondary traumatic stress^a^ Psychological distress^a^ Emotion^a^ Empathic stress^a^ Burnout^a^ Resilience^a^ Vicarious resilience^a^ Vicarious posttraumatic growth^a^ Compassion satisfaction^a^ Compassion fatigue^a^ Coping^a^ Coping behaviours^a^ Coping mechanisms^a^ Coping strategies^a^ Client inspired hope^a^	Support^a^ Support networks^a^ Organisational support^a^ Training^a^ Supervision^a^ Shadowing^a^ Peer support^a^ Self-care^a^ Mindfulness^a^ Reflection^a^

*Note*. ^a^Used as a wildcard in order to broaden the search by finding derived words that start with the same letters.

#### Databases searched

Seven academic databases (PsycINFO/Articles, MEDLINE, Lexisnexis, Ovid Full Text Journals, Sage Journals, Taylor and Francis Online, Wiley Online Library) and one nonacademic database (Google Scholar) were searched. These high-profile, often-used databases were chosen due to their wide coverage of multiple disciplines and perspectives, their accessibility, and the quality of information provided (Brophy & Bawden, 2005). PsycINFO and PsycArticles were searched concurrently to reduce the number of duplicated papers.

#### Data abstraction

**Figure 1.**
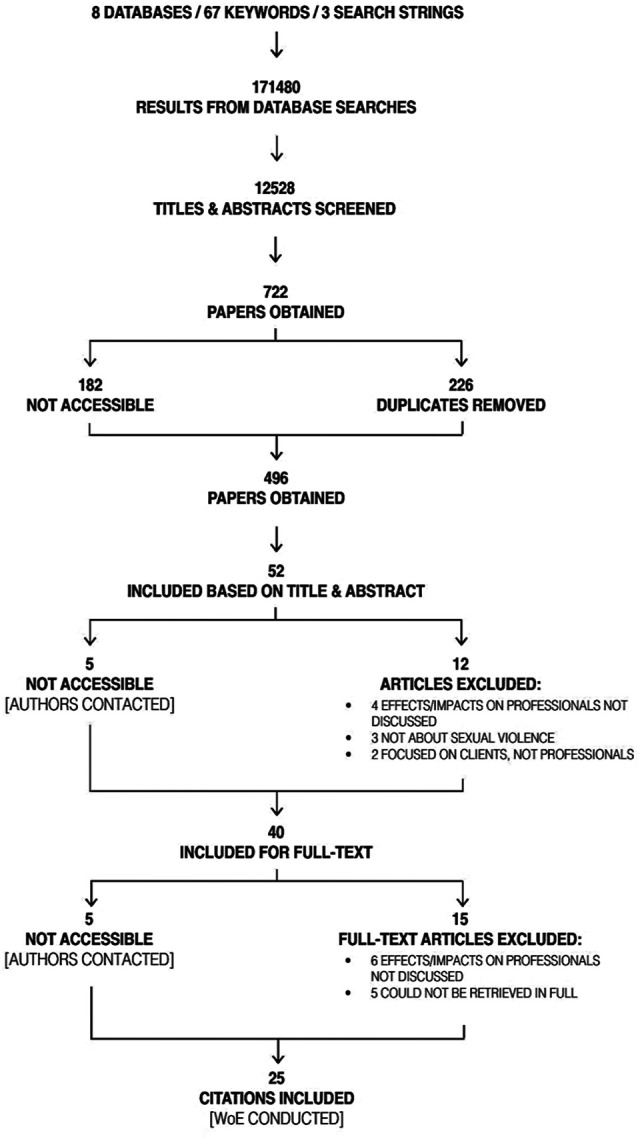
Deductive map of the search process.

The search was conducted in two parts. Firstly, databases were independently searched by one of the researchers, with titles and abstracts screened to fit the inclusion and exclusion criteria. Fifty-two papers were chosen as appropriate once duplicates were removed. Secondly, the three researchers discussed the possible papers resulting in a total of 40 studies included for full-text read. Five papers were excluded as they could not be obtained. Ultimately, 25 papers met the inclusion criteria and were analyzed. Figure 1 presents a detailed deductive map of the search process.

#### Weight of Evidence (WoE) coding and data synthesis

Included papers were then evaluated using a simplified WoE approach, as proposed by Evidence for Policy and Practice Information and Co-ordinating Centre (Gough, 2007). This allowed for a consistent and objective analysis of various studies regardless of the methodologies and statistical analyses they implemented. This method has been successfully used in other research (Gekoski et al., 2015).

On a scale of *low* (1), *medium* (2), and *high* (3), each author evaluated each paper independently in terms of *confidence* in the paper itself to respond to its research questions using appropriate analyses and methodologies and *relevance* of the paper to present results that addressed the purpose of the REA. Papers were scored lower in quality if they were not peer reviewed, if they were not judged as having answered their hypotheses, or if they employed overly complicated or overly simple designs, and vice versa for higher quality studies. Papers were judged as poorly relevant to this REA if they did not fully answer the REA questions and if there was uncertainty about the professionals’ roles with sexual violence victims, and vice versa for highly relevant studies. To strengthen the quality of the findings, a rigorous three-step assessment triangulation was conducted (Denzin, 1978; Patton, 1990). Firstly, three of the included articles were randomly chosen, independently evaluated by each of the authors, and then discussed. Secondly, 10 more articles were chosen in alphabetical order and were independently scored by the authors and then discussed. Finally, the remaining articles were independently assessed and scored without further conferencing. Average confidence and relevance scores were then computed for each article. Studies with lower assessments were given less weight in the synthesis and vice versa for studies with higher assessments. The articles were read in depth and synthesized. Commonalities, patterns, and themes in their findings were extracted using thematic analysis: codes were generated, common themes were searched for, found themes were reviewed, a thematic map was created, and the themes were defined and named before summarizing the findings (Braun & Clarke, 2006).

## Findings

### Overall Characteristics

The majority of the papers (*N* = 20) were published peer-reviewed research articles and five were publicly available doctoral theses. The majority were studies conducted in the United Kingdom (*N* = 9) and in the United States (*N* = 9), and a smaller amount (*N* = 4) in Australia. In terms of methodology, most (*N* = 12) used qualitative designs or eight quantitative designs. Most studies (*N* = 22) were of medium to high methodological quality (low = 3, medium = 11, high = 11), and most (*N* = 15) were of high relevance to this REA (low = 5, medium = 5, high = 15).

The papers included participants from multiple professions, who held a variety of roles such as victim advocates, social workers, police officers, mental health care staff, and administrative staff. Sample sizes ranged from 6 to 564, with a mean sample size of 80 participants. Women (*N* = 1,612) were the majority of participants in all studies (*N* = 23). Only 10 studies mentioned the participant’s ethnicity, and in all but one, the majority of participants self-identified as Caucasian/White.

### Effects of Working With Victim-Survivors of Sexual Violence on Professionals’ Well-Being

The effects of working with victim-survivors of sexual violence were clearly divided between being negative or positive. These will be considered below.

#### Negative impacts

Various degrees of negative and disruptive effects on staff’s personal and professional well-being were reported by all 25 papers. A strong emphasis on the negatives of this work is demonstrated in the literature. This makes intuitive sense as the focus of many of the papers is to understand the impact on staff, find ways to avoid burnout, and reduce trauma in staff. The impacts were never stand-alone, but in a perpetual cycle of interrelationships, translating into behavioral changes which then further sustained the cognitive, emotional, and somatic distress experienced.

##### Trauma symptoms

In the papers which used psychometric testing (e.g., Secondary Traumatic Stress Scale [STSS]; Professional Quality of Life Scale [ProQOL]) to assess staff’s VT or STS levels and symptomatology, there were more studies reporting significant signs of traumatization (Dutton et al., 2017; Kreinath, 2019; Newman et al., 2019) than those suggesting minimal to no symptoms (Makadia et al., 2017; Rostron & Furlonger, 2017).

Negative, disruptive effects of working with victims of sexual violence were also prevalent in staff’s interviews. Avoidance, intrusive thoughts, nightmares, flashbacks, and persistent visual imagery of client’s abuse were often present (Brend et al., 2020; Hunt, 2018; Joubert et al., 2017; Parkes et al., 2019a).

##### Perceptions of safety and loss of trust

Overall, the most commonly reported changes were to the safety and trust cognitive schemas. In 11 studies, participants reported hypervigilance and hyperarousal (Albaek et al., 2020; Fedele, 2018; Kreinath, 2019). Common effects of these altered cognitions included paranoia and increased feelings of concern and responsibility toward personal safety, family and friends’ safety, as well as their clients’ (even when no longer working with them). Staff found themselves changing their day-to-day behaviors in order to take extra precautions, such as no longer taking taxis, following reports of clients being sexually abused by taxi drivers. For those professionals who had children, especially if they worked with child victims or adult survivors of child sexual abuse, their parenting styles became overprotective and controlling (Parkes et al., 2019a; Rostron & Furlonger, 2017). Some staff, particularly males, reported constantly reevaluating their interactions with their own children (Parkes et al., 2019a) or their family in general (Brend et al., 2020). Professionals also became increasingly suspicious and distrustful of other’s intentions, with an emphasis on a loss of trust in males generally, family members, or males around children (Coleman, 2018; Parkes et al., 2019a; Taylor et al., 2019). Importantly, and perhaps a unique effect of working with sexual violence, experiencing increased suspiciousness about other’s intentions negatively affected professionals’ intimate relationships with spouses or partners through intrusive thoughts or visual imagery, inability to enjoy sexual relationships, decreased libido, lack of trust, increased self-consciousness, and general altered behaviors toward partners (Parkes et al., 2019a; Rostron & Furlonger, 2017). It sometimes resulted in a reduced desire or curiosity to attempt any future relationships due to feeling unsafe at the possibility of perhaps meeting an abuser (Kreinath, 2019; Taylor et al., 2019).

The lack of trust also extended to undermine their belief in the criminal justice system (Massey et al., 2019; Nixon, 2019), which led to emotional distress, such as feelings of hopelessness or powerless in their roles and feeling guilty when not able to help or support clients to the extent they would wish to (Albaek et al., 2020; Brend et al., 2020; Nixon, 2019). The hierarchical structure of the criminal justice system, or the mental health system in some cases (Albaek et al., 2020), meant that professionals found it difficult to do their job. They often faced ethical and professional dilemmas about their role and the usefulness or necessity of their role (Backe, 2018; Javaid, 2017). Faced with these issues, staff felt stressed and unable to cope with the demands of the job, their sense of self-worth, confidence, and self-efficacy as professionals were diminished, felt disempowered and meaningless in their job, as well as experiencing constant doubt and ruminations over decisions taken due to no longer perceiving themselves as skilled to a high standard (Albaek et al., 2020; Brend et al., 2020; Gatuguta et al., 2019; O’Dwyer et al., 2019). This effect was exacerbated when they lacked clear guidance, guidelines, or resources (Backe, 2018; Kreinath, 2019), when there was an overreliance on procedures, and when they did not feel that the organizations they worked for would support their decisions (Albaek et al., 2020).

##### Emotional and psychological distress

The volume of work and having to empathetically engage with client’s stories and feelings left professionals experiencing emotional and psychological distress. Most commonly, they felt fearful, depressed, anxious, sad, angry, upset, horrified, fatigued or drained, and frustrated (Dutton et al., 2017; Hunt, 2018; O’Dwyer et al., 2019). Sometimes they wanted to get away from clients and looked ahead with dread, finding it hard to motivate themselves to come to work (Brend et al., 2020; Parkes et al., 2019a). Sometimes staff found it difficult to keep clear boundaries between work life and personal life (Massey et al., 2019; Taylor et al., 2019), and these effects translated into altered social relationships, with them becoming more irascible, short-tempered, and generally more “down” when with family and friends (Brend et al., 2020; Hunt, 2018). Additionally, professionals could feel alone and isolated in their roles and duties. Due to the nature of the job, they felt that they could not speak to family or friends about this (Parkes et al., 2019a). Feelings of isolation were more prevalent and distressing in those professionals who received little support and validation from managers (Brend et al., 2020), or were working out-of-hour or remote shifts with limited or no possibility to interact with colleagues and superiors (Massey et al., 2019). Furthermore, changes to their frame of reference were found, with some staff finding it difficult to reconcile their view of the work with the abuse stories heard (Albaek et al., 2020; Rostron & Furlonger, 2017). Due to the distressing content they were exposed to, as well as the general lack of resolutions for the victims, staff developed a more pessimistic and cynical world view (Parkes et al., 2019a), or they became desensitized to the trauma (Massey et al., 2019). Moreover, several studies found that staff experienced difficulty sleeping or insomnia (Dutton et al., 2017), crying or increased tearfulness (Parkes et al., 2019a; Rostron & Furlonger, 2017), particularly when faced with a perceived injustice such as when perpetrators were acquitted (Nixon, 2019).

Several studies reported significant levels of burnout in staff, whether this was perceived/self-reported or psychometrically tested (Backe, 2018; Javaid, 2017; Taylor et al., 2019). This appeared to be the result of a complex interplay of workplace factors, the nature and volume of the job, as well as personality traits. A circular relationship was found between cumulative emotional stress, lack of workplace understanding and support leading to feelings of disempowerment, and heightened burnout in helpline workers (Taylor et al., 2019). For example, average levels of burnout were found in one study with specialist police officers, which were positively associated with their degree of dispositional empathy, their client group (working with adult victims), and number of years spent in the specialist service working with sexual violence (Turgoose et al., 2017). This research controlled for numbers of years spent in the police force overall and found that it was the specific work with sexual violence which affected staff, leading to a significant relationship between STS, CF, and burnout in the sample. High workloads, particularly, depleted professionals’ energy and resilience, thus increasing the risk of burnout and other traumatic symptoms (Parkes et al., 2019b). Usually through the mediating role of burnout in the job, cumulative negative impacts on staff, without taking actions to address them, lead to either a change in roles, such as working with a client group perceived as less difficult, or resulted in staff leaving the job, despite enjoying it (Rostron & Furlonger, 2017; Taylor et al., 2019). However, through attending a retreat focused on holistic healing and general well-being, burnout levels were decreased in a group of U.S. professionals (Dutton et al., 2017).

#### Positive impacts

Alongside the negative impact, research in this area identified that there are positive experiences of sexual violence work on the staff’s personal and professional lives. However, as much of the focus of the work in this area is on how to support staff, there is less focus on the rewards of working in the sexual violence field.

##### Satisfaction and fulfillment

In some papers, it was clear that despite the hardships of the role, in terms of the nature and volume of work in unsupportive organizations and wider systems, professionals took pride in doing an important, meaningful, and necessary job (Gatuguta et al., 2019; Hunt, 2018; Parkes et al., 2019b). Some saw the role as a “calling” and felt privileged to be able to be part of the clients’ lives and help them (Brend et al., 2020). The positives of the work were a main reason for them staying in the role (Nixon, 2019). Professionally, participants experienced increased compassion satisfaction levels (Brend et al., 2020; Kreinath, 2019); professional growth through consistently improving their clinical skills and being more self-aware (Coleman, 2018; Hunt, 2018); developing a sense of perspective, fulfillment, satisfaction, and achievement, or feeling empowered, happy, and hopeful in their role, particularly when able to help the clients overcome the trauma or reach satisfactory results in courts (Brend et al., 2020; Nixon, 2019; Parkes et al., 2019a); and posttraumatic growth (Frey et al., 2017).

##### Improved personal lives

Working with victims of sexual violence also had positive effects in their personal lives, such as feeling more pleasure and contentment with their family life and spousal relationships (Brend et al., 2020; Coleman, 2018); having improved relationships with friends and family, particularly with other women (Taylor et al., 2019); developing more confidence, assertiveness, and personal resilience (Dutton et al., 2017; Frey et al., 2017); and being more aware of stereotypes and biases around sexual violence offenses and victims, including decreases in their sexist views (Brend et al., 2020).

### Factors Influencing Ability to Cope With Impacts

Professionals’ ability to deal with the negative impacts of their work was dependent on two types of factors: individual factors, relating to the professionals themselves, and organizational, relating to the work environment and job characteristics. These factors either diminished or increased the negative effects of working with survivors or helped professionals experience more positive aftermaths of the work, either on a personal or on a professional level. These intrinsic and situational factors, considered below, were interrelated, each affecting the other.

#### Individual factors

Sociodemographic variables (age, income, educational level) and individual characteristics (ethnicity, gender identity, gender expression, sexual identity, sexual orientation) did not predict STS levels (Choi, 2017; Makadia et al., 2017), nor did they influence the development of positive effects of working with survivors of sexual trauma, such as vicarious posttraumatic growth or compassion satisfaction (Frey et al., 2017). However, for some, being self-aware, knowing themselves and their limits as professionals had a buffering effect on VT (Brend et al., 2020; Nixon, 2019). Others had lower levels of STS and experienced vicarious posttraumatic growth if they had higher levels of personal resilience and psychological empowerment, and if they felt that they made a difference in their clients’ lives (Brend et al., 2020; Frey et al., 2017). Denkinger et al. (2018) looked at professionals’ attachment styles and found positive relationships between VT and preoccupied attachment styles, while a negative relationship was found with secure attachment styles. In a sample of female crisis workers holding more radical feminist beliefs, and strongly perceiving themselves as feminists resulted in increased vulnerability to VT, while, simultaneously, holding stronger social feminist beliefs related to more VG (Fedele, 2018). Studies that investigated personal history of trauma as an individual factor to professionals’ well-being had mixed results. In a study of U.S.-based professionals, Hunt (2018) found that, due to the nature of the work, professionals’ personal trauma was resurfaced and possibly impacted on their retraumatization. For correctional and forensic health staff, a history of both single- and multiple- personal trauma increased their susceptibility to VT and were especially correlated with the VT subscale of avoidance, while having a history of multiple traumas increased all subscales of hyperarousal, intrusion, and avoidance, with symptoms higher in severity compared to those professionals without personal trauma (Newman et al., 2019). It appears that having a personal history of trauma may not be enough. Denkinger et al. (2018) found that for professionals working with refugees, who themselves had experienced sexual trauma and fleeing their home countries, having personally experienced flight, together with other types of trauma and working many hours of direct (face-to-face) contact with the clients, contributed to the development of secondary trauma. Identifying with or having experienced similar events as the client’s trauma were risk factors. On the other hand, professionals perceiving their own personal trauma as resolved and overcome was an important buffering factor (Brend et al., 2020), with personal trauma sometimes even promoting the development of vicarious resilience (Frey et al., 2017).

#### Organizational factors

Organizational factors and work environment appear to have a greater influence than individual factors. Generally, studies focused on the support available, including supervision and guidance, workload and caseload characteristics, including perceived control over them, and the type of clients professionals worked with. While what was meant by “support” was not clearly defined in all studies, it was evident that perceiving the organization, the work environment, including colleagues, and the management as organizationally and operationally supportive impacted on professionals’ well-being and the quality of the services they provided to their clients (Choi, 2017; O’Dwyer et al., 2019; Turgoose et al., 2017).

Organizational support enhanced professionals’ ability to cope with the distressing nature of the job and its effects. Having the possibility of quality informal supervision (i.e., talking to colleagues and debriefing with them) or peer support, either as a group or on an individual basis, and overall being part of an environment high in team spirit and camaraderie, were consistently seen as protective factors in reducing vulnerability to STS or the severity of traumatic and negative symptoms, including burnout (Brend et al., 2020; Choi, 2017; Taylor et al., 2019). These also promoted personal resilience, self-confidence, and posttraumatic growth even after controlling for other factors (Frey et al., 2017). Choi (2017) referred to this team cohesiveness and support as “shared power,” which, in their group of social workers, was the only organizational factor, among several researched, which was negatively associated with STS. Additionally, professionals found it beneficial and protective to have their distressing experiences recognized and normalized through support from peers and superiors (Parkes et al., 2019b).

Organizational support included providing formal supervision. Studies indicated that receiving formal supervision reduced secondary trauma, or protected against it, and decreased work-related stress and burnout (Kreinath, 2019; Massey et al., 2019; Nixon, 2019). Through formal supervision, professionals had a designated space and time to explore their emotions about work and personal life, which could otherwise be transferred to the client. Additionally, to discuss cases receiving reassurance about their decisions, skills, and roles, thus reducing self-doubt and rumination, and instead increasing the overall quality of their work. On the other hand, lack of supervision and support heightened overall negative impacts, stress, and burnout (Albaek et al., 2020; Backe, 2018; Kreinath, 2019; Makadia et al., 2017). However, in Denkinger et al.’s (2018) study receiving supervision was not related to VT, suggesting that quality of supervision is paramount. Several studies supported the idea of a change from quantity to quality supervision, with it being most effective if it was needs based and in the format desired by the professionals (Brend et al., 2020; Massey et al., 2019). Ideally, formal supervision would be done regularly with a qualified external person both in one-to-one and group-based format (Taylor et al., 2019).

When combined with a lack of understanding and support from superiors, working with conflicting guidelines led to staff experiencing ambiguity and ethical dilemmas about their profession and the quality of their work (Joubert et al., 2017; Nixon, 2019). Poor internal guidance left staff questioning their self-worth and their actions, being doubtful about their skills and the help they were providing (Albaek et al., 2020), feeling helpless and disempowered in their role (Backe, 2018; O’Dwyer et al., 2019). For example, staff at a rape crisis helpline felt overwhelmed and anxious in having to manage negative interactions with clients without having received training, resources, and guidance in how to do so (Backe, 2018). When resources were also scarce, lack of support and guidance could further lead to propagation of sexist beliefs, rape-supportive myths, and unsafe practices, which could even hinder police investigations of rape allegations (Gatuguta et al., 2019; O’Dwyer et al., 2019). On the other hand, a supportive environment and receiving guidance protected against negative impacts and secondary traumatization (Nixon, 2019).

In supporting victims, PSWSV appeared to be managing high workloads and caseloads (with little to no control over them) in high-pressure environments. This pressure could be due to the organization’s inner structure and demands or because of the nature of working with clients navigating the criminal justice system. Overall, it led to the negative impacts already outlined. These reduced professionals’ ability to ensure clear boundaries between work and personal life, which affected their psychological and emotional well-being, and their social behavior and relationships. There also seemed to be conflict and tension between organizational or system (e.g., Criminal Justice System) pressures to “get the job done” through any means possible and with little regard for self-care, which increased traumatic symptoms (Backe, 2018; Javaid, 2017; Kreinath, 2019).

Several studies found that large caseloads were positively associated with secondary traumatization and negative impacts (Denkinger et al., 2018; Makadia et al., 2017; Turgoose et al., 2017). Importantly, amount of time spent in direct, face-to-face contact with clients was a contributing factor to increased negative impacts (Benuto et al., 2018). Even when staff were not experiencing secondary traumatization, their sense of safety and trust, a symptom commonly associated with PTSD, could be seen (Fedele, 2018). Some professionals described the job as “relentless” and felt overwhelmed due to supporting a large caseload in a fast-paced environment (Joubert et al., 2017). There were some indications that supporting victims during night shifts (Massey et al., 2019) and when working remotely or from home (Taylor et al., 2019) was more emotionally draining, and staff found it harder to cope with their feelings as well as the clients’ demands.

### Minimizing the Impacts and Facilitating Well-being

There were a number of mechanisms at both organizational and individual level, which studies found minimized the negative impacts and facilitated well-being among PSWSVs. These are considered below.

#### Organizational level

Being part of a supportive organization and team was a vital protective factor that helped minimize negative effects, while promoting positive outcomes of the work (Choi, 2017; Frey et al., 2017; Hunt, 2018; Parkes et al., 2019b). It was important that the organizations took proactive measures in reducing the impacts and promoting well-being. Psychological impacts were less disruptive when staff felt validated and empowered by their peers and, particularly, managers (Albaek et al., 2020; Brend et al., 2020; Taylor et al., 2019). Having a designated space, allocated time, and a qualified professional for both informal and formal supervision was also crucial (Coleman et al., 2018; Hunt, 2018; Kreinath, 2019). Some professionals suggested that even if no measures were taken by the organization, they wanted recognition of the distressing nature of the work and the normalization of experiencing negative impacts (Massey et al., 2019; Parkes et al., 2019b).

Several studies commented on the importance of training as a significant method of supporting staff and helping them cope. There were two levels of training: firstly, a pragmatic, skills-based one, related to job requirements, such as guidance and resources in how to manage difficult clients, how to appropriately use professional guidelines, or specific knowledge about sexual violence (Backe, 2018; Gatuguta et al., 2019; Nixon, 2019). Skill acquisition through high-quality training was consistently seen as a factor minimizing secondary traumatization for various professionals (Brend et al., 2020; Hunt, 2018; Makadia et al., 2017), leading to increased confidence and ability to offer better support to clients. Based on their individual job requirements, as well as areas of insecurity or confusion in the role, professional themselves desired more overall operational guidance, specialist, needs-based and ongoing training, together with better resources, such as supervision and manageable workloads (Brend et al., 2020; O’Dwyer et al., 2019; Parkes et al., 2019a). There was also the need for “emotional” training, which included providing professionals with individual tools, such as coping mechanisms, to manage the effects and enhance the positives of doing the role. Staff wanted to learn about possible negative impacts of working with sexual violence victims, how to manage their emotions, how to identify warning signs in themselves, and how to reduce distressing effects (Backe, 2018; Taylor et al., 2019; Turgoose et al., 2017). Managerial and senior staff also benefited from training in how to better support staff and promote their well-being (Turgoose et al., 2017), and staff wanted more knowledgeable managers (Parkes et al., 2019a, 2019b)

#### Individual level

Coping mechanisms and self-care strategies were vital factors promoting staff’s psychological and physical well-being, both at work and in private, also enabling them to do their jobs better (Denkinger et al., 2018; Nixon, 2019; Rostron & Furlonger, 2017). Adaptive coping mechanisms, restorative practices, and positive self-help strategies or activities were particularly useful, both in the short- and long term. These included individualistic strategies such as practicing mindfulness, spending time in nature, doing physical exercise or playing sports, having hobbies, engaging in recreational activities, such as reading, and taking part in activist projects and social strategies such as spending time with family and friends, talking about the impacts with members of the social group, and having an active social life.

Self-care strategies could also be implemented while at work, for example taking breaks or time off, going for walks, seeking peer support and supervision, psychological distancing, or meditating (Kreinath, 2019). Also important for the promotion of overall psychological well-being were staff being self-aware of the impacts and actively trying to build resilience, accepting that not every client can be helped and reminding themselves of the good job they were doing, accepting their professional limitations and consistently trying to expand their skills and knowledge, maintaining healthy boundaries between work and personal life, having a family life as stress-free as possible, and overall having good social relationships and support systems in their life (Albaek et al., 2020; Nixon, 2019).

On the other hand, maladaptive coping strategies, significantly more present in police samples, were a superficial short-term “fix” and with the potential, in the long-term, to exacerbate negative impacts both at work and in staff’s personal lives possibly through reducing staff’s ability to maintain clear boundaries (O’Dwyer et al., 2019; Parkes et al., 2019b; Rostron & Furlonger, 2017). They included avoidance-based (e.g., putting off the work, procrastinating or focusing on more menial tasks; deliberate blocking of intrusive thoughts; doing the work automatically, without cognitive engagement or awareness; vagueness; withdrawal), detachment-based (e.g., denial and pretending the abuse is not real; emotional detachment and “turning off” empathy) and process-driven strategies (e.g., looking at the abuse as factual and from a legal point of view, disregarding the victim involved in it).

## Discussion

This REA is the only of its kind to review recent publications investigating the impacts and effects of working with victims of sexual violence on professionals, factors affecting staff’s coping ability, and individual and organizational factors facilitating well-being. Table 2 presents a summary of the critical findings.

**Table 2.** Summary of Critical Findings.

Impacts and effects	Both negative and positive impacts Impacts were interconnected and affected both personal and professional life Negative impacts: intrusive thoughts, rumination, hypervigilance, hyperarousal, lack of trust, anger, frustration, sadness, anxiety, guilt, hopelessness, helplessness, loneliness and alienation, disrupted social relationships (including sexual life), self-doubt, feelings of powerlessness, burnout, compassion fatigue, insomnia, diminished ability to cope with the work, behavioral changes (e.g., overprotectiveness, changed routines; leaving work). Positive effects: feelings of hope, happiness, empowerment, and validation; increased assertiveness and personal resilience, self-awareness and confidence; posttraumatic growth; professional growth; improved relationships; increased specialist knowledge and diminished stereotypical opinions about victims of sexual violence.
Factors influencing ability to cope with impacts	Ability to cope with the demands of the work was dependent on interconnected organizational and intrinsic factors Increased ability to cope when higher levels of organizational support through supervision, peer support, guidance and understanding from supervisors, ensuring clear guidelines, and having manageable workloads with sufficient resources (e.g. time) High pressure, high workloads, and lack of support, together with caseload characteristics diminished ability to cope, increased negative impacts and decreased quality of the help provided to clients Being self-aware, having personal resilience, feeling empowered in the role and feeling that they made a difference in the clients’ lives increased ability to cope and positive impacts of the job Having a personal history of trauma both increased negative impacts and helped with posttraumatic growth Sociodemographic variants did not influence effects
Minimizing the impacts and facilitating well-being	Minimizing mechanisms present at organizational- and individual-level Receiving support and guidance from the organization, supervisors, and colleagues were protective factors Receiving regular needs-based training which developed both specialist skills and ability to deal with the psychological impacts of the work was desired and seen as a main factor facilitating overall well-being in the workplace Using adaptive coping mechanisms while at work and in the personal life, having a good social support system, being self-aware of own personal and professional limits, putting the role in perspective and praising oneself for doing an important job while accepting that not all clients can be helped, as well as actively building specialist knowledge and personal resilience, were important factors promoting well-being and minimizing negative impacts

This is a new and emerging area of research, and as such, there is a deficit of literature. International literature was used, which brings with it strengths and weaknesses. As different countries have extremely different health systems, criminal justice systems, and access to counseling or Employee Assistance Programs, it is almost impossible to compare a worker supporting rape victims in Britain with one in Kenya. Cultural and religious differences coupled with differing levels of support available to the victim will vary by country, putting far more pressure on the professional in a country where there is little social care or health care to provide peripheral support. Additionally, the level of support for the professional will also vary to a similar extent and access to support within and outside of the organization will be very different in different national contexts.

Staff predominantly experienced negative impacts when working in unsupportive environments, with limited training, resources, peer support and supervision, while managing high workloads. For some, the impact of this work was dependent on client group and case type. Distress levels and coping ability were also associated with individual factors such as resilience, specialist knowledge levels, beliefs, not enough time spend on self-care and coping strategies, and individual personality type. There were mixed results regarding the influence of a personal history of trauma on professional’s ability to cope with the work.

For an organization to support its staff, it must recognize the negative consequences of working with acutely distressed clients, especially in the area of sexual violence (Clarke, 2011). It is imperative that sexual violence organizations develop safe spaces for their staff to talk freely about their emotional struggles. This will allow staff to express themselves honestly and reduce the perception that they need to always portray a personal competence and infallibility to colleagues and superiors instead of asking for help (Leary & Kowalski, 1990). Organizations must also play an active role in providing their staff the necessary support and opportunities for skill and resilience development (Baird & Jenkins, 2003; Jackson et al., 2007). For example, professionals who feel more satisfied with their workplace and perceive their work as meaningful tend to have lower levels of trauma or burnout (Collins & Long, 2003; Ludick & Figley, 2017). In the studies included in this REA, feelings of satisfaction, competence, empowerment, self-efficacy, and “making a difference” protected against STS and burnout. Such results are in line with earlier research (Bell et al., 2003; Figley, 2002b). If no proactive measures were taken to minimize distress and promote well-being, the cumulated negative effects could (in addition to other elements) lead staff to leave work. Indeed, absenteeism and intentions to leave work are common in staff experiencing traumatic symptoms and job burnout (Maslach et al., 2001).

There are 25 studies included in this review. While this is an encouraging amount given the specificity of the field of PSWSV, increased national and international research would make it easier to draw stronger assertions and generalizations across cultures, professions, countries, and systems. In addition, the current number of available articles means that generalizations were made across organizations that had little in common apart from a client base that included people who had experienced sexual violence.

Some studies in this REA relied on very small participant numbers as PSWSV can be a difficult professional group to access. They tend to have large caseloads and there are not many of them nationally. As such, the level of empirical rigor that can be found in some studies is not possible with this population.

One such cultural difference is attitudes to women and equality. As yet, we have not achieved worldwide equality for women and so staff in some countries will be faced with larger difficulties around helping and supporting in cases of sexual violence or intimate partner violence (IPV) than in others countries. Rape and IPV are not illegal in all countries. Therefore, attitudes to victims, support for victims, and funding for this sort of work will be affected by the social context of where the study was carried out. This presents a difficulty in the literature where not all workers are supporting clients in a country where what has happened to them is illegal. It is highly likely that staff in these countries will lack knowledge, skills, resources, and training in working with victims, and they may be struggling to cope in unsupportive criminal justice and wider social and health systems (Gatuguta et al., 2019).

The rates of PTSD shown in sexual violence workers may also have been caused by the international nature of the literature. Different countries vary in their diagnostic systems. Canada and the United States use the *Diagnostic and Statistical Manual of Mental Disorders, Fifth Edition* that recognizes the cumulative effect of trauma. Other countries use the *International Classification of Diseases* *-11* that has different diagnostic categories. In the United States, medical insurance companies usually require a diagnosis to fund therapy, so a worker who is struggling with things they have seen at work will typically be diagnosed with a mental health condition for the therapy to be funded. In many other countries, this is not the case and as such diagnostic levels of conditions such as PTSD differ by country.

In this REA, literature pertaining to different types of sexual violence workers were included. One of the founders of work in this area, Patricia Yancy Martin (2005) found different emotional responses in workers with differing levels of engagement and roles with sexual violence clients. For example, rape crisis staff and police officers had different emotional responses to responding to sexual violence. As such, the findings of the REA may be affected by combining “workers in the field of sexual violence” regardless of role. With an increase in literature into this topic, it will allow for more nuanced interpretations of the literature and a better understanding of the impact of working in the sexual violence field depending on role.

However, some issues do seem to be universal. For example, the literature shows a lack of training for staff on issues such as effective ways to deal with the negative feelings that arise from working with highly traumatized people, dealing with cases which end in an unsatisfactory way, dealing with the clients from those “badly resolved” cases (and their emotions, frustrations, and their place in society and the criminal justice system). There is a clear need for formal, compulsory training on these topics to support staff.

It is clear from studies predating this REA, as well as studies included, that sexual violence workers rely on the rewarding parts of the work to buffer against the impacts (Brend et al., 2020; Martin, 2005). Meaning is found in the successes and rewards of the work, and these positive elements of the work sustain staff through the more difficult times.

Very few of the 25 studies in this REA included administrative staff in the participant pool (Backe, 2018; Kreinath, 2019). Therefore, it was difficult to draw conclusions regarding the effects on staff who worked with sexual violence victims in different ways. For example, many offices have a general phone number and an administrator may be the first point of contact for very distressed clients, before transferring them to trained workers. That administrator will usually get no supervision or recognition for the emotional element of their work. In studies where the participants were professionals working in diverse environments (e.g., acute ward, prison, domestic violence shelter, police) (Javaid, 2017; Kreinath, 2019; Newman et al., 2019; O’Dwyer et al., 2019), there is an added level of complexity. It is even more difficult to ascertain the traumatic effects of working with this population from the effects of the workplace. It becomes unclear whether any trauma found in workers is due to cumulative exposure to workplace trauma (e.g., threats of physical abuse) or from the client-facing work with traumatized individuals (including sexual violence trauma). As such, this unique population requires and deserves further research.

The importance of fully understanding the impact of working with sexual violence was highlighted in studies that showed that the consequences of secondary traumatization could be felt in the professional–client relationship. When left unchecked, they can lead to disrupted alliances, violations of professional boundaries, and inadequate or inappropriate reactions from staff (Zimering et al., 2003). This means that the impact of vicarious trauma is far reaching and doubly concerning. As such, we need to understand what can be done to avoid it, reduce it, treat it, and support people with it. The effect of working with traumatized people is not a direct or simple one. This is demonstrated in the work of authors such as Frey et al. (2017) whose research pointed out that work-related and intrapersonal vicarious posttraumatic growth and growth in staffs’ private lives were interconnected and could not be untangled.

### Methodological Issues

Most studies were of high methodological quality. Nonetheless, some common methodological problems were noticed, and these could be addressed in future research. Firstly, the concepts investigated such as VT or STS were not clearly defined, which led to them being used interchangeably. For example, Newman et al. (2019) investigated VT as a symptom of PTSD, when it could be argued that it is in fact a concept of its own, related more to STS, yet different due to altering an individual’s cognitive schemas as a result of exposure to cumulative trauma, as well as including trauma symptoms (McCann & Pearlman, 1990). Some studies looked at posttraumatic growth, while others looked at the same, or similar, or subscale symptoms under the term of vicarious posttraumatic growth or vicarious growth. Others investigated concepts as a whole, for example STS, while others only their subscales, most of the time lacking depth in relating impacts and effects to their wider conceptual definitions. Combined, these issues led to a lack of uniformity in reporting findings. Overall ambiguity in the findings and uncertainty of what they mean poses practical issues in developing universal assessment tools and methods to help professionals cope with the work. Additionally, it poses issues for future research through overlapping concepts that limit the reliability and validity of the conclusions to be drawn from empirical research, thus diminishing the possible advice to be given for practice. The need for future research to address these points has been consistently mentioned in previous literature (Branson, 2019; Quitangon & Evces, 2015).

Secondly, there were several sample issues. In terms of gender and ethnic diversity, overall, there were significantly more female participants than males or other genders, and in all but one paper, the majority, or all participants, self-identified as Caucasian /White. While it is likely that more women may work with victims of sexual violence than men, there is greater ethnic diversity in PSWSV than is represented in the papers in this REA. As such, the current findings are difficult to generalize to professionals who do not belong to these groups. It is advised that future research addresses the important issue of diversity. For example, one study in this REA (O’Dwyer et al., 2019) indicated that male health workers found it particularly difficult and uncomfortable to address sexual violence against women and lacked the confidence to do so. Additionally, in some studies, participants were part of one or maybe two organizations, and their recruitment, or even survey distribution, was done via their managers. Participants could have thus provided more socially desirable answers and been reluctant to truthfully comment on negative workplace factors. Combined with the overall overreliance on qualitative designs and self-reporting, results could have also been affected by a self-serving bias.

Lastly, it would have been beneficial for more studies to include quantifiable measures of type and amount of contact professionals had with the clients, especially when they worked in environments in which survivors of sexual violence were just one client group out of many. Several studies in this REA found that amount of exposure through face-to-face contact predicted the severity of the impacts (Denkinger et al., 2018). In order to provide better advice for practice, future studies need to consider this factor.

### Implications for Practice

The studies included in this REA make it very clear that it is not enough for organizations to simply attempt to minimize the impacts of working with victims of sexual violence, but they need to actively promote and teach how to create positive effects. This responsibility falls to the organization and trainers to overtly instill an ethos of self-care, collegiately and acceptance of emotions evoked by the work done. Biases and rape myths in staff must be addressed in an attempt to minimize them, and training and supervision should not only be offered, it should be required. In organizations, time and space need to be prioritized for activities such as supervision and training because sexual violence staff have such large caseloads, trying to keep up with day-to-day work, which often makes other tasks feel like luxury, not necessity. Organizations must provide specialist, external and professional supervision, which meets the needs of the staff. Similarly, training must be specialist and of quality, and it must be about what the professionals in that organization feel would be helpful. It’s important to have the technical knowledge, but sexual violence staff also need to learn the necessary skills they feel they are lacking or learn skills to manage the emotional work they do.

Although there is little work in this area, it does seem that there needs to be better support for the staff who are often overlooked, such as administration, trainers, and interpreters (Backe, 2018; Denkinger et al., 2018; Kreinath, 2019). As mentioned above, this group is often exposed to high levels of emotion and to distressing tasks in the reports they are transcribing, or in the phone calls they are taking. They do not, however, usually qualify for supervision or support from their organization.

In many countries, organizations have a duty of care to their staff. It is a requirement to create a work environment that is not harmful to the employee. Just as a manual laborer may need a hard hat to stay safe at work, a sexual violence worker needs holistic training, good quality regular supervision, a cohesive supportive team, and clear guidelines from both their employer and the government.

### Limitations

Some databases yielded limited results, with no publications that fit the inclusion criteria based on titles and abstracts. Moreover, studies published after the end of the search (January 31, 2020) will have been missed by this REA. Thus, given the rapid flow of publications in the area of impacts of working with sexual trauma, as well as the methodology of the REA, it may not have been possible to find and assess all the available studies, especially those currently in press. Five studies meeting the inclusion criteria could not be retrieved in full. Additionally, no gray literature and publications in languages other than English were searched. While this REA tried to be as comprehensive as possible, relevant research may have still been missed. As this is an emerging area, there was only a limited number of articles to draw from. This is not a topic that has received a great deal of attention to date. As such, conclusions had to be drawn from a limited number of articles.

## Conclusion

Research into the effects of working with traumatized people is still in its infancy. Although there has been some interest into the effects of working in certain professions such as psychotherapy and social work, there is little research in the area of working with people who have experienced sexual violence. This article draws together the limited existing research in this area. A strong picture emerges to show that working with victims of sexual violence strongly impacts professionals. However, this relationship is possibly more complex than originally thought with both negative and positive effects on staff’s psychological well-being, quality of their work, and their personal life. The literature in this REA shows that working in the sexual violence field impacts workers and as such they require support from their organizations, other staff members, and social networks. The importance of training and supervision was highlighted in the literature, as working with this highly traumatized population requires skill, resilience, coping strategies, and support. The limited literature on this topic demonstrates that far more research is needed to properly understand how to best support people who do, what could be considered, the hardest work in the caring professions.

### Implication for Practice

- Clearer internal practice guidelines within the organization.
- More resources and better allocation of resources to meet the needs of the individual and the team.
- Normalization of negative impacts of the work.
- Provide staff with space and time to be heard and supported.
- Increased quantity and quality of formal supervision which meets the needs of the individual and the team.
- Increased quantity and quality of training.
- Training for staff and supervisors/managers in order to increase specialist knowledge, skills, and ability to cope with the psychological and emotional demands of the work.
- Organizations, including supervisors/managers, to actively promote staff’s well-being, improve team cohesiveness and support.
- Staff to be supported by the organization working within the system, particularly the Criminal Justice System.

### Implication for Policy

- Clearer professional practice guidelines.
- Systemic support and funding for the organizations.
- More job positions within organizations in order to reduce individual workload and pressure and increase quality of the time and help professionals are able to provide each client with.

### Implication for Future Research

- Consistent terminology.
- Clearly defined terminology and research aims within the studies.
- More diverse samples in terms of gender and ethnicity.
- Collect data on social, economic, educational, and cultural background.
- Collect data and take into consideration job variables (e.g., amount of face-to-face work; client type and client complexity; workload).
- Control as much as possible the cumulative effect of other variants within the role (e.g., working with verbally abusive clients; working within mental health).
- Greater care taken when collecting data in order to reduce self-serving bias and/or socially desirable responding.
- More studies using quantitative and mixed-methods designs.
- Focus on researching at-work adaptive coping mechanisms.
- Focus on researching how to enhance the positive impacts of the job in order to provide clearer guidance to the organizations.

## Note

1. The term “professional” is used throughout this article to denote any staff member, in paid or volunteering positions, who works with traumatized clients in any professional capacity (e.g., therapists, interpreters, crisis workers, and administrative staff).

## References

[bibr1-15248380211016024] Albaek A. U. Binder P. E. Milde A. M. (2020). Plunging into a dark sea of emotions: Professionals’ emotional experiences addressing child abuse in interviews with children. Qualitative Health Research, 30(8), 1212–1224. 10.1177/1049732318825145 30674238

[bibr2-15248380211016024] Backe E. L. (2018). A crisis of care: The politics and therapeutics of a rape crisis hotline. Medical Anthropology Quarterly, 32(4), 463–480. 10.1111/maq.12463 29968935

[bibr3-15248380211016024] Baird S. Jenkins S. R. (2003). Vicarious traumatization, secondary traumatic stress, and burnout in sexual assault and domestic violence agency staff. Violence and Victims, 18(1), 71–86. 10.1891/vivi.2003.18.1.71 12733620

[bibr90-15248380211016024] Bakker A. B. Heuven E. (2006). Emotional dissonance, burnout, and in-role performance among nurses and police officers. International Journal of Stress Management, 13(4), 423–440. 10.1037/1072-5245.13.4.42

[bibr4-15248380211016024] Bell H. Kulkarni S. Dalton L. (2003). Organizational prevention of vicarious trauma. Families in Society, 84(4), 463–470. 10.1606/1044-3894.131

[bibr5-15248380211016024] Benuto L. T. Newlands R. Ruork A. Hooft S. Ahrendt A. (2018). Secondary traumatic stress among victim advocates: Prevalence and correlates. Journal of Evidence-Informed Social Work, 15(5), 494–509. 10.1080/23761407.2018.1474825 29856279

[bibr6-15248380211016024] Blair D. T. Ramones V. A. (1996). Understanding vicarious traumatization. Journal of Psychosocial Nursing and Mental Health Services, 34(11), 24–30. 10.3928/0279-3695-19961101-15 8923347

[bibr7-15248380211016024] Brady J. L. Guy J. D. Poelstra P. L. Brokaw B. F. (1999). Vicarious traumatization, spirituality, and the treatment of sexual abuse survivors: A national survey of women psychotherapists. Professional Psychology: Research and Practice, 30(4), 386–393. 10.1037/0735-7028.30.4.386

[bibr8-15248380211016024] Branson D. C. (2019). Vicarious trauma, themes in research, and terminology: A review of literature. Traumatology, 25(1), 2. 10.1037/trm0000161

[bibr9-15248380211016024] Braun V. Clarke V. (2006). Using thematic analysis in psychology. Qualitative Research in Psychology, 3(2), 77–101. 10.1191/1478088706qp063oa

[bibr10-15248380211016024] Brend D. M. Krane J. Saunders S. (2020). Exposure to trauma in intimate partner violence human service work: A scoping review. Traumatology, 26(1), 127–136. 10.1037/trm0000199

[bibr11-15248380211016024] Bride B. E. (2007). Prevalence of secondary traumatic stress among social workers. Social Work, 52(1), 63–70. 10.1093/sw/52.1.63 17388084

[bibr12-15248380211016024] Bride B. E. Hatcher S. S. Humble M. N. (2009). Trauma training, trauma practices, and secondary traumatic stress among substance abuse counselors. Traumatology, 15(2), 96–105. 10.1177/1534765609336362

[bibr13-15248380211016024] Briere J. Jordan C. E. (2004). Violence against women: Outcome complexity and implications for assessment and treatment. Journal of Interpersonal Violence, 19(11), 1252–1276. 10.1177/0886260504269682 15534329

[bibr14-15248380211016024] Brophy J. Bawden D. (2005). Is Google enough? Comparison of an internet search engine with academic library resources. Aslib Proceedings, 57(6), 498–512. 10.1108/00012530510634235

[bibr15-15248380211016024] Burke R. J. (1994). Stressful events, work-family conflict, coping, psychological burnout, and well-being among police officers. Psychological Reports, 75(2), 787–800. 10.2466/pr0.1994.75.2.787 7862787

[bibr16-15248380211016024] Choi G. Y. (2011). Secondary traumatic stress of service providers who practice with survivors of family or sexual violence: A national survey of social workers. Smith College Studies in Social Work, 81(1), 101–119. 10.1080/00377317.2011.543044

[bibr17-15248380211016024] Choi G. Y. (2017). Secondary traumatic stress and empowerment among social workers working with family violence or sexual assault survivors. Journal of Social Work, 17(3), 358–378. 10.1177/1468017316640194

[bibr18-15248380211016024] Clarke J. (2011). Working with sex offenders: Best practice in enhancing practitioner resilience. Journal of Sexual Aggression, 17(3), 335–355. 10.1080/13552600.2011.583781

[bibr19-15248380211016024] Clemans S. E. (2004). Life changing: The experience of rape-crisis work. Affilia, 19(2), 146–159. 10.1177/0886109903262758

[bibr20-15248380211016024] Coleman A. M. Chouliara Z. Currie K. (2018). Working in the field of complex psychological trauma: A framework for personal and professional growth, training, and supervision. Journal of Interpersonal Violence, 36(5–6), 1–25. Advance online publication. 10.1177/0886260518759062 29557712

[bibr21-15248380211016024] Collins S. Long A. (2003). Working with the psychological effects of trauma: Consequences for mental health-care workers—A literature review. Journal of Psychiatric and Mental Health Nursing, 10(4), 417–424. 10.1046/j.1365-2850.2003.00620.x 12887633

[bibr22-15248380211016024] Cosden M. Sanford A. Koch L. M. Lepore C. E. (2016). Vicarious trauma and vicarious posttraumatic growth among substance abuse treatment providers. Substance Abuse, 37(4), 619–624. 10.1080/08897077.2016.1181695 27163485

[bibr23-15248380211016024] Cunningham M. (2003). Impact of trauma work on social work clinicians: Empirical findings. Social Work, 48(4), 451–459. 10.1093/sw/48.4.451 14620102

[bibr24-15248380211016024] Daniels S. J. (2016). Working with the trauma of rape and sexual violence: A guide for professionals. Jessica Kingsley Publishers.

[bibr25-15248380211016024] Davies P . (2003). The magenta book. Guidance notes for policy evaluation and analysis. Chapter 2: What do we already know? Cabinet Office.

[bibr26-15248380211016024] Denkinger J. K. Windthorst P. Rometsch-Ogioun El Sount C Blume M. Sedik H. Kizilhan J. I. Gibbons N. Pham P. Hillebrecht J. Ateia N. Nikendei C. Zipfel S. Junne F . (2018). Secondary traumatization in caregivers working with women and children who suffered extreme violence by the “Islamic State.” Frontiers in Psychiatry, 9, 234. 10.3389/fpsyt.2018.00234 29922186 PMC5996169

[bibr27-15248380211016024] Denzin N. K. (1978). The research act: A theoretical introduction to sociological methods. McGraw Hill.

[bibr28-15248380211016024] Dutton M. A. Dahlgren S. Franco-Rahman M. Martinez M. Serrano A. Mete M. (2017). A holistic healing arts model for counselors, advocates, and lawyers serving trauma survivors: Joyful heart foundation retreat. Traumatology, 23(2), 143–152. 10.1037/trm0000109

[bibr29-15248380211016024] Elwood L. S. Mott J. Lohr J. M. Galovski T. E. (2011). Secondary trauma symptoms in clinicians: A critical review of the construct, specificity, and implications for trauma-focused treatment. Clinical Psychology Review, 31(1), 25–36. 10.1016/j.cpr.2010.09.004 21130934

[bibr30-15248380211016024] Fedele K . (2018). An investigation of factors impacting vicarious traumatization and vicarious posttraumatic growth in crisis workers: Vicarious exposure to trauma, feminist beliefs, and feminist self-labeling [Doctoral dissertation, University of Akron]. https://etd.ohiolink.edu/!etd.send_file?accession=akron1519564198322496&disposition=inline

[bibr31-15248380211016024] Figley C. R. (1995). Compassion fatigue as secondary traumatic stress disorder: An overview. In Figley C. R. (Ed.), Compassion fatigue: Coping with secondary traumatic stress disorder in those who treat the traumatized (pp. 1–20). Brunner/Mazel Publishers.

[bibr32-15248380211016024] Figley C. R. (1996). Review of the compassion fatigue self-test. In Stamm B. H. (Ed.), Measurement of stress, trauma, and adaptation (pp.127–130). Sidran Press.

[bibr33-15248380211016024] Figley C. R. (2002a). Compassion fatigue: Psychotherapists’ chronic lack of self care. Journal of Clinical Psychology, 58(11), 1433–1441. 10.1002/jclp.10090 12412153

[bibr34-15248380211016024] Figley C. R . (Ed.). (2002b). Treating compassion fatigue. Brunner-Routledge.

[bibr35-15248380211016024] Folkman S. Moskowitz J. T. (2004). Coping: Pitfalls and promise. Annual Review of Psychology, 55(1), 745–774. 10.1146/annurev.psych.55.090902.141456 14744233

[bibr36-15248380211016024] Frey L. L. Beesley D. Abbott D. Kendrick E. (2017). Vicarious resilience in sexual assault and domestic violence advocates. Psychological Trauma: Theory, Research, Practice, and Policy, 9(1), 44–51. 10.1037/tra0000159 27268097

[bibr37-15248380211016024] Gatuguta A. Colombini M. Seeley J. Soremekun S. Devries K. (2019). Supporting children and adolescents who have experienced sexual abuse to access services: Community health workers’ experiences in Kenya. Child Abuse & Neglect, 104244. Advance online publication. 10.1016/j.chiabu.2019.104244 31882066

[bibr38-15248380211016024] Gekoski A. Gray J. M. Horvath M. A. Edwards S. Emirali A. Adler J. R . (2015). ‘What Works*’* in reducing sexual harassment and sexual offences on public transport nationally and internationally: A rapid evidence assessment. British Transport Police and Department for Transport. https://eprints.mdx.ac.uk/id/eprint/15219

[bibr39-15248380211016024] Gough D. (2007). Weight of evidence: A framework for the appraisal of the quality and relevance of evidence. Research Papers in Education, 22(2), 213–228. 10.1080/02671520701296189

[bibr40-15248380211016024] Hesse A. R. (2002). Secondary trauma: How working with trauma survivors affects therapists. Clinical Social Work Journal, 30(3), 293–309. 10.1023/A:1016049632545

[bibr41-15248380211016024] Horvath M. A. H. Massey K. (2018). The impact of witnessing other people’s trauma: The resilience and coping strategies of members of the faculty of forensic and legal medicine. Journal of Forensic and Legal Medicine, 55, 99–104. 10.1016/j.jflm.2018.02.012 29486433

[bibr42-15248380211016024] Howard A. R. H. Parris S. Hall J. S. Call C. D. Razuri E. B. Purvis K. B. Cross D. R. (2015). An examination of the relationships between professional quality of life, adverse childhood experiences, resilience, and work environment in a sample of human service providers. Children and Youth Services Review, 57, 141–148. 10.1016/j.childyouth.2015.08.003

[bibr43-15248380211016024] Hunt T . (2018). Professionals’ perceptions of vicarious trauma from working with victims of sexual trauma [Doctoral dissertation, Walden University, MN, United States]. https://scholarworks.waldenu.edu/dissertations/5879/

[bibr44-15248380211016024] Jackson D. Firtko A. Edenborough M. (2007). Personal resilience as a strategy for surviving and thriving in the face of workplace adversity: A literature review. Journal of Advanced Nursing, 60(1), 1–9. 10.1111/j.1365-2648.2007.04412.x 17824934

[bibr45-15248380211016024] Jahnke S. A. Poston W. S. C. Haddock C. K. Murphy B. (2016). Firefighting and mental health: Experiences of repeated exposure to trauma. Work: A Journal of Prevention, Assessment & Rehabilitation, 53(4), 737–744. 10.3233/WOR-162255 26890595

[bibr46-15248380211016024] Javaid A . (2017). “Walking on egg shells”: Policing sexual offences against men. The Police Journal, 90(3), 228–245. 10.1177/0032258X16677357

[bibr47-15248380211016024] Joubert E. M. Van Aswegen E. J. Havenga Y. D . (2017). An emancipatory model for nurses working with gender-based violence in a semi-rural area in Tshwane [Doctoral dissertation, Sefako Makgatho Health Sciences University, South Africa]. https://www.semanticscholar.org/paper/An-emancipatory-model-for-nurses-working-with-in-a-Joubert-Aswegen/97ae11d3c0a13384f7ecd9fbe8090e454b6a1a6c

[bibr48-15248380211016024] Kang X. Fang Y. Li S. Liu Y. Zhao D. Feng X. Wang Y. Li P. (2018). The benefits of indirect exposure to trauma: The relationships among vicarious posttraumatic growth, social support, and resilience in ambulance personnel in China. Psychiatry Investigation, 15(5), 452. 10.30773/pi.2017.11.08.1 29695152 PMC5976003

[bibr49-15248380211016024] Kessler R. C. Aguilar-Gaxiola S. Alonso J. Benjet C. Bromet E. J. Cardoso G. Degenhardt L. de Girolamo G. Dinolova R. V. Ferry F. Florescu S. Gureje O. Maria Haro J. Huang Y. Karam E. G. Kawakami N. Lee S. Lepine J. Levinson D. Navarro-Mateu F.…Koenen K. C . (2017). Trauma and PTSD in the WHO world mental health surveys. European Journal of Psychotraumatology, 8(sup5), 1353383, 10.1080/20008198.2017.1353383 29075426 PMC5632781

[bibr50-15248380211016024] Kessler R. C. Sonnega A. Bromet E. Hughes M. Nelson C. B. (1995). Posttraumatic stress disorder in the national comorbidity survey. Archives of General Psychiatry, 52(12), 1048–1060, 10.1001/archpsyc.1995.03950240066012 7492257

[bibr51-15248380211016024] Kreinath R. S . (2019). Secondary and vicarious traumatization among domestic violence shelter staff [Doctoral dissertation, Wichita State University, United States]. https://www.semanticscholar.org/paper/Secondary-and-vicarious-traumatization-among-staff-Kreinath/c6adda03acb6cdd61c3d75c120bae5a8635242ea

[bibr52-15248380211016024] Leary M. R. Kowalski R. M. (1990). Impression management: A literature review and two-component model. Psychological Bulletin, 107(1), 34–47. 10.1037/0033-2909.107.1.34

[bibr53-15248380211016024] Ludick M. Figley C. R. (2017). Toward a mechanism for secondary trauma induction and reduction: Reimagining a theory of secondary traumatic stress. Traumatology, 23(1), 112–123. 10.1037/trm0000096

[bibr54-15248380211016024] Luthar S. S. Cicchetti D. (2000). The construct of resilience: Implications for interventions and social policies. Development and Psychopathology, 12(4), 857–885. 10.1017/S0954579400004156 11202047 PMC1903337

[bibr55-15248380211016024] Makadia R. Sabin-Farrell R. Turpin G . (2017). Indirect exposure to client trauma and the impact on trainee clinical psychologists: Secondary traumatic stress or vicarious traumatization? Clinical Psychology & Psychotherapy, 24(5), 1059–1068. 10.1002/cpp.2068 28124447

[bibr56-15248380211016024] Manning D. Majeed-Ariss R. Mattison M. White C. (2019). The high prevalence of pre-existing mental health complaints in clients attending Saint Mary’s sexual assault referral centre: Implications for initial management and engagement with the independent sexual violence advisor service at the centre. Journal of Forensic and Legal Medicine, 61, 102–107. 10.1016/j.jflm.2018.12.001 30551033

[bibr57-15248380211016024] Martin P. Y. (2005). Rape work: Victims, gender and emotions in organization and community context. Routledge.

[bibr58-15248380211016024] Martinussen M. Richardsen A. M. Burke R. J. (2007). Job demands, job resources, and burnout among police officers. Journal of Criminal Justice, 35(3), 239–249. 10.1016/j.jcrimjus.2007.03.001

[bibr59-15248380211016024] Maslach C . (2003). Burnout: The cost of caring. Malor Books.

[bibr60-15248380211016024] Maslach C. Leiter M. P. (2006). Burnout. In Rossi A. M. Perrewe P. L. Sauter S. L. (Eds.), Stress and quality of working life: Current perspectives in occupational health (pp. 42–49). IAP.

[bibr61-15248380211016024] Maslach C. Schaufeli W. B. Leiter M. P. (2001). Job burnout. Annual Review in Psychology, 52, 397–442. 10.1146/annurev.psych.52.1.397 11148311

[bibr62-15248380211016024] Mason F. Lodrick Z. (2013). Psychological consequences of sexual assault. Best Practice & Research Clinical Obstetrics & Gynaecology, 27(1), 27–37. 10.1016/j.bpobgyn.2012.08.015 23182852

[bibr63-15248380211016024] Massey K. Horvath M. A. H. Essafi S. Majeed-Ariss R. (2019). Staff experiences of working in a sexual assault referral centre: The impacts and emotional tolls of working with traumatised people. The Journal of Forensic Psychiatry & Psychology, 30(4), 686–705. 10.1080/14789949.2019.1605615

[bibr64-15248380211016024] McCann C. M. Beddoe E. McCormick K. Huggard P. Kedge S. Adamson C. Huggard J. (2013). Resilience in the health professions: A review of recent literature. International Journal of Wellbeing, 3(1), 60–81. 10.5502/ijw.v3i1.4

[bibr65-15248380211016024] McCann I. L. Pearlman L. A. (1990). Vicarious traumatization: A framework for understanding the psychological effects of working with victims. Journal of Traumatic Stress, 3(1), 131–149. 10.1007/BF00975140

[bibr66-15248380211016024] Newman C. Eason M. Kinghorn G. (2019). Incidence of vicarious trauma in correctional health and forensic mental health staff in New South Wales, Australia. Journal of Forensic Nursing, 15(3), 183–192. 10.1097/JFN.0000000000000245 31259816

[bibr67-15248380211016024] Nixon M. A. (2019). A qualitative exploration of therapists’ experience of working therapeutically pre-trial within the crown prosecution service guidelines with adult clients who have reported sexual violence [Doctoral dissertation, University of Chester, United Kingdom]. https://chesterrep.openrepository.com/bitstream/handle/10034/622176/Nixon%20Madelyn%20SO7101%20Dissertation.pdf?sequence=1&isAllowed=y

[bibr68-15248380211016024] O’Dwyer C. Tarzia L. Fernbacher S. Hegarty K. (2019). Health professionals’ experiences of providing care for women survivors of sexual violence in psychiatric inpatient units. BMC Health Services Research, 19(1), 1–9. 10.1186/s12913-019-4683-z 31727056 PMC6857150

[bibr69-15248380211016024] Office for National Statistics. (2020, July 17). Crime in England and Wales: Year ending March 2020. file:///D:/User%20Files/YO/Downloads/Crime%20in%20England%20and%20Wales%20year%20ending%20March%202020.pdf

[bibr70-15248380211016024] Pack M. (2013). Vicarious traumatisation and resilience: An ecological systems approach to sexual abuse counsellors’ trauma and stress. Sexual Abuse in Australia and New Zealand, 5(2), 69–76.

[bibr71-15248380211016024] Parkes R. Graham-Kevan N. Bryce J. (2019a). You don’t see the world through the same eyes anymore: The impact of sexual offending work on police staff. The Police Journal, 92(4), 316–338. 10.1177/0032258X18812515

[bibr72-15248380211016024] Parkes R. Graham-Kevan N. Bryce J . (2019b). I put my “police head” on: Coping strategies for working with sexual offending material. The Police Journal, 92(3), 237–263. 10.1177/0032258X18808294

[bibr73-15248380211016024] Patton M. Q. (1990). Qualitative evaluation and research methods. Sage Publishers.

[bibr74-15248380211016024] Pearlman L. A. Saakvitne K. W. (1995). Treating therapists with vicarious traumatization and secondary traumatic stress disorders. In Figley C. (Ed.), Compassion fatigue: Coping with secondary traumatic stress disorder in those who treat the traumatized (pp. 150–177). Brunner/Mazel.

[bibr75-15248380211016024] Quitangon G. Evces M. R. (Eds.). (2015). Vicarious trauma and disaster mental health: Understanding risks and promoting resilience. Routledge.

[bibr76-15248380211016024] Rizkalla N. Zeevi-Barkay M. Segal S. P. (2017). Rape crisis counseling: Trauma contagion and supervision. Journal of Interpersonal Violence, 36, 1–24, 10.1177/0886260517736877 29294964

[bibr77-15248380211016024] Robinson D. (2018, October 04). Employers must look after your mental health too [Blog post]. https://www.miphealth.org.uk/home/news-campaigns/Features/legal-eye-employers-must-look-after-mental-health.aspx

[bibr78-15248380211016024] Rostron M. S. Furlonger B. (2017). A preliminary investigation of vicarious traumatisation among forensic medical examiners of sexual assault. Journal of Counselling Profession, 1(1), 37–48. http://www.hkpca.org.hk/download/74039/

[bibr79-15248380211016024] Sabatini Gutierrez N. (2018). Finding our voices: Understanding sexual identity and relational well-being in female therapists working with survivors of sexual trauma [Doctoral dissertation, Alliant International University, San Diego, CA, United States]. https://search.proquest.com/openview/1672bb46a725fafc9d858abfff74ef8e/1?pq-origsite=gscholar&cbl=18750&diss=y

[bibr80-15248380211016024] Smith B. W. Tooley E. M. Christopher P. J. Kay V. S. (2010). Resilience as the ability to bounce back from stress: A neglected personal resource? The Journal of Positive Psychology, 5(3), 166–176. 10.1080/17439760.2010.482186

[bibr81-15248380211016024] Taylor A. K. Gregory A. Feder G. Williamson E . (2019). We’re all wounded healers: A qualitative study to explore the well-being and needs of helpline workers supporting survivors of domestic violence and abuse. Health & Social Care in the Community, 27(4), 856–862. 10.1111/hsc.12699 30592098

[bibr82-15248380211016024] Turgoose D. Glover N. Barker C. Maddox L. (2017). Empathy, compassion fatigue, and burnout in police officers working with rape victims. Traumatology, 23(2), 205–213. 10.1037/trm0000118

[bibr83-15248380211016024] Ullman S. E. (2016). Sexual revictimization, PTSD, and problem drinking in sexual assault survivors. Addictive Behaviors, 53, 7–10. 10.1016/j.addbeh.2015.09.010 26414205 PMC4679471

[bibr84-15248380211016024] United Nations Women (2017, November 24). Eight countries that are making historic changes to ensure no woman or girl is left behind. Medium. https://medium.com/we-the-peoples/eight-countries-that-are-making-historic-changes-to-ensure-no-woman-or-girl-is-left-behind-dc753920549f

[bibr85-15248380211016024] Vandenberghe A. Hendriks B. Peeters L. Roelens K. Keygnaert I. (2018). Establishing sexual assault care centres in Belgium: Health professionals’ role in the patient-centred care for victims of sexual violence. BMC Health Services Research, 18(1), 807. 10.1186/s12913-018-3608-6 30348151 PMC6196455

[bibr86-15248380211016024] Varker T. Forbes D. Dell L. Weston A. Merlin T. Hodson S. O’Donnell M. (2015). Rapid evidence assessment: Increasing the transparency of an emerging methodology. Journal of Evaluation in Clinical Practice, 21(6), 1199–1204. 10.1111/jep.12405 26123092

[bibr87-15248380211016024] Vrklevski L. P. Franklin J. (2008). Vicarious trauma: The impact on solicitors of exposure to traumatic material. Traumatology, 14(1), 106–118. 10.1177/1534765607309961

[bibr88-15248380211016024] Zeidner M. Hadar D. Matthews G. Roberts R. D. (2013). Personal factors related to compassion fatigue in health professionals. Anxiety, Stress & Coping, 26(6), 595–609. 10.1080/10615806.2013.777045 23614527

[bibr89-15248380211016024] Zimering R. Munroe J. Gulliver S. B. (2003). Secondary traumatization in mental health care providers. Psychiatric Times, 20(4), 20–28.

